# Cytokine and chemokine map of peripheral specific immune cell subsets in Parkinson’s disease

**DOI:** 10.1038/s41531-023-00559-0

**Published:** 2023-07-25

**Authors:** Si-Si Jiang, Yi-Ling Wang, Qiu-Han Xu, Lu-Yan Gu, Rui-Qing Kang, Wen-Yi Yang, Bao-Rong Zhang, Jun Tian, Jia-Li Pu

**Affiliations:** Department of Neurology, the Second Affiliated Hospital, School of Medicine, Zhejiang University, Hangzhou, Zhejiang China

**Keywords:** Parkinson's disease, Neuroimmunology

## Abstract

Peripheral immune cells play a vital role in the development of Parkinson’s disease (PD). However, their cytokine and chemokine secretion functions remain unclear. Therefore, we aimed to explore the cytokine and chemokine secretion functions of specific immune cell subtypes in drug-naïve patients with PD at different ages of onset. We included 10 early-onset and 10 late-onset patients with PD and age-matched healthy controls (HCs). We used mass cytometry to select specific immune cell subsets and evaluate intracellular cytokine and chemokine expression. Statistical tests included *t*-tests, analysis of variance, bivariate correlation analysis, and linear regression analysis. Compared with HCs, patients with PD exhibited significantly decreased intracellular pro-inflammatory cytokines and chemokines in selected clusters (e.g., tumor necrosis factor (TNF)-α, interleukin (IL)−8, IL-1β, and CC-chemokine ligand (CCL)17). Specific cytokines and cell clusters were associated with clinical symptoms. TNF-α played an important role in cognitive impairment. Intracellular TNF-α levels in the naïve CD8^+^ T-cell cluster C16 (CD57^−^ naïve CD8^+^ T) and natural killer (NK) cell cluster C32 (CD57^−^ CD28^−^ NK) were negatively correlated with Montreal Cognitive Assessment scores. The C16 cluster affected cognitive function and motor symptoms. Increased TNF-α and decreased interferon-γ expression in C16 correlated with increased Unified Parkinson’s Disease Rating Scale III scores in patients with PD. In summary, we developed a more detailed cytokine and chemokine map of peripheral specific CD8^+^ T cell and NK cell subsets, which revealed disrupted secretory function in patients with PD and provided unique clues for further mechanistic exploration.

## Introduction

Parkinson’s disease (PD) is a neurodegenerative disorder with an annual incidence of 8–18.6 new cases per 100,000 and a prevalence of 1.7% in people over 65 years of age in China^1^. Unfortunately, current treatments are still ineffective in alleviating dopaminergic neuron dysfunction, since the pathogenesis of PD remains unclear^2^. In recent years, there has been increasing evidence that neuroimmunity plays a vital role in the development of PD, and immune cell dysfunction, in particular, has received widespread attention^3^. Given the considerable bidirectional crosstalk between peripheral inflammation and neuroimmunity^4^, the measurement of immune markers in peripheral blood may be an easier and less invasive way to monitor immune responses in PD.

A cluster of α-synuclein-specific T cells, which are activated by specific antigenic epitopes and release interferon (IFN)-γ, interleukin (IL)-4, and IL-10, are present prior to clinical diagnosis of PD^5,6^. Furthermore, naïve CD4^+^ T cells in patients with PD preferentially differentiate into Th1 cells in peripheral blood and secrete large amounts of IFN-γ and tumor necrosis factor (TNF)-α^7,8^. In vitro, experiments have shown that phosphorylated α-synuclein epitopes stimulate CD4^+^ T cells to upregulate IL-17A expression^9^. In addition, chemokines have been found to play a central role in the regulation of neuroinflammatory responses, mediating immune cell migration through the blood–brain barrier, which leads to inflammatory neural damage^10^. Cerebrospinal fluid (CSF) of patients with Lewy body dementia exhibits upregulated expression of C-X-C motif chemokine receptor 4 (CXCR4), as well as the CXCR4 ligand, C-X-C motif chemokine ligand 12 (CXCL12), in CD4^+^ T cells, which are associated with neuroaxonal damage^9^. The role of various immune cells in PD is increasingly recognized, and screening for these cells and elucidating their function will contribute to our understanding of PD-specific immune status. Most studies have focused on CD4^+^ T cells, whereas the functions of CD8^+^ T cells and other immune cells, such as NK cells, require further elucidation.

A previous study by our group, which explored the frequency of specific immune cell subtypes in the peripheral blood of drug-naïve patients with PD, found decreased effector CD8^+^ T cells and NK cells with reduced cytotoxicity, which were associated with age, unified Parkinson’s disease rating scale (UPDRS) scores, and mini-mental state examination (MMSE) scores^11^. The mechanisms of peripheral immune cell changes in PD, which are related to functional changes rather than to the frequency of peripheral immune cells, require in-depth exploration. Therefore, we aimed to explore the functions, especially cytokine and chemokine secretion functions, of the previously identified specific immune cell subtypes in drug-naïve patients with PD at different ages of onset, to elucidate the relationship between the peripheral immune response and PD and identify reliable biomarkers for early diagnosis.

## Results

### Mass cytometry of subsets and intracellular cytokine and chemokine expression in specific CD8^+^ T cells and NK cells

**Fig. 1:**
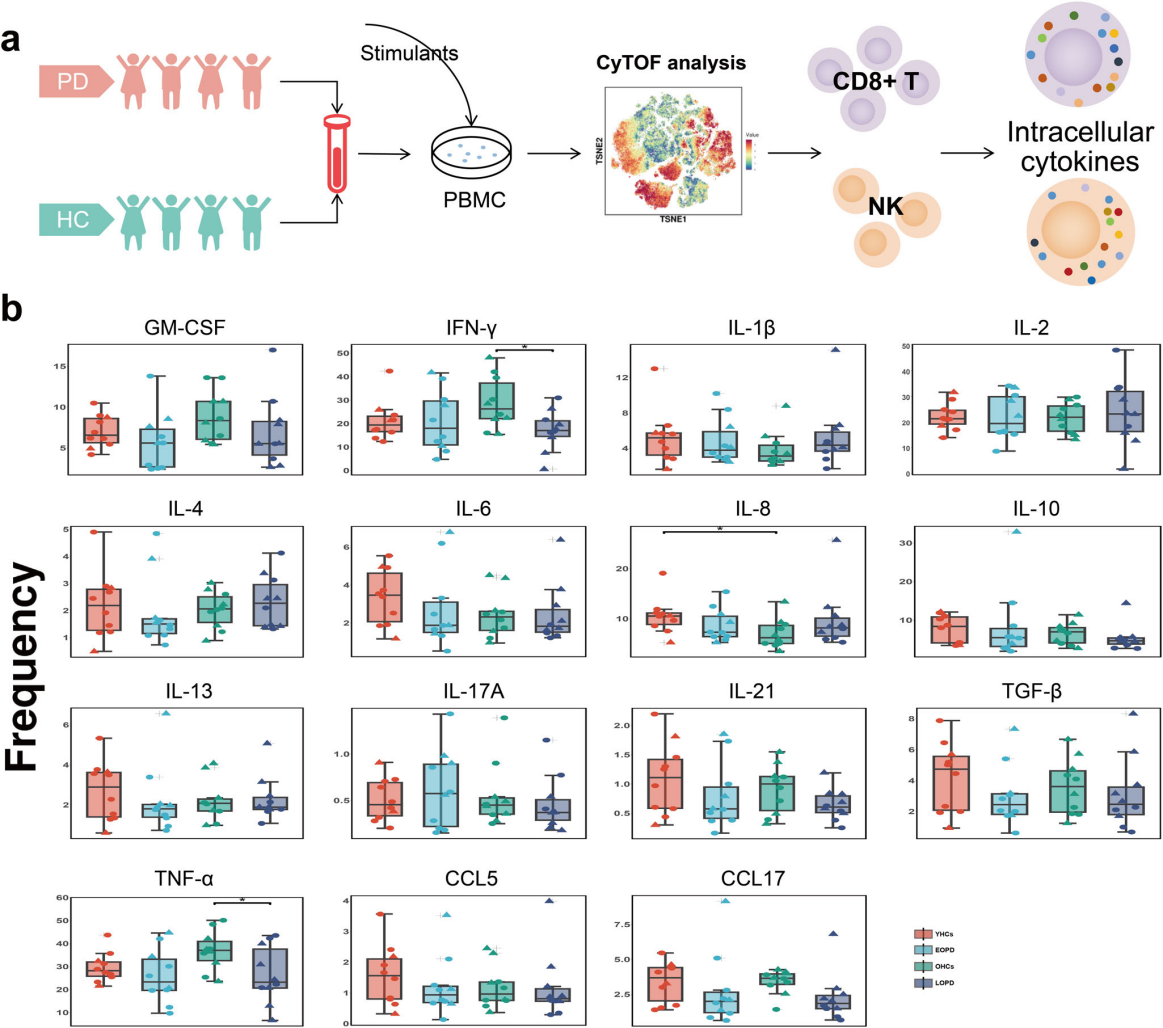
Differences in cytokine and chemokine expression in specific immune cell subsets. **a** Graphical abstract. We used CyTOF analysis to select specific immune cell clusters and evaluate intracellular cytokine and chemokine expression. **b** IFN-γ and TNF-α are significantly decreased in LOPD compared with that in OHCs. ANOVA with Bonferroni correction was used to test statistical significance between groups (*p < 0.05, **p < 0.01, ***p < 0.001). Error bars show the mean ± standard error of the mean (SEM). The box-and-whisker plot was presented as median, 1st quartile, and 3rd quartile of the box, the 95th and 5th percentile for the upper and lower whisker, "+" for the outliners. △ = male; ○ = female. OHCs older adult healthy controls, EOPD early-onset PD, HCs healthy controls, LOPD late-onset PD, PD Parkinson’s disease, t-SNE t-distributed stochastic neighbor embedding, YHCs young healthy controls, IL interleukin, CCL17 C–C motif chemokine ligand 17, TGF-β transforming growth factor-β, GM-CSF granulocyte-macrophage colony-stimulating factor.

**Fig. 2:**
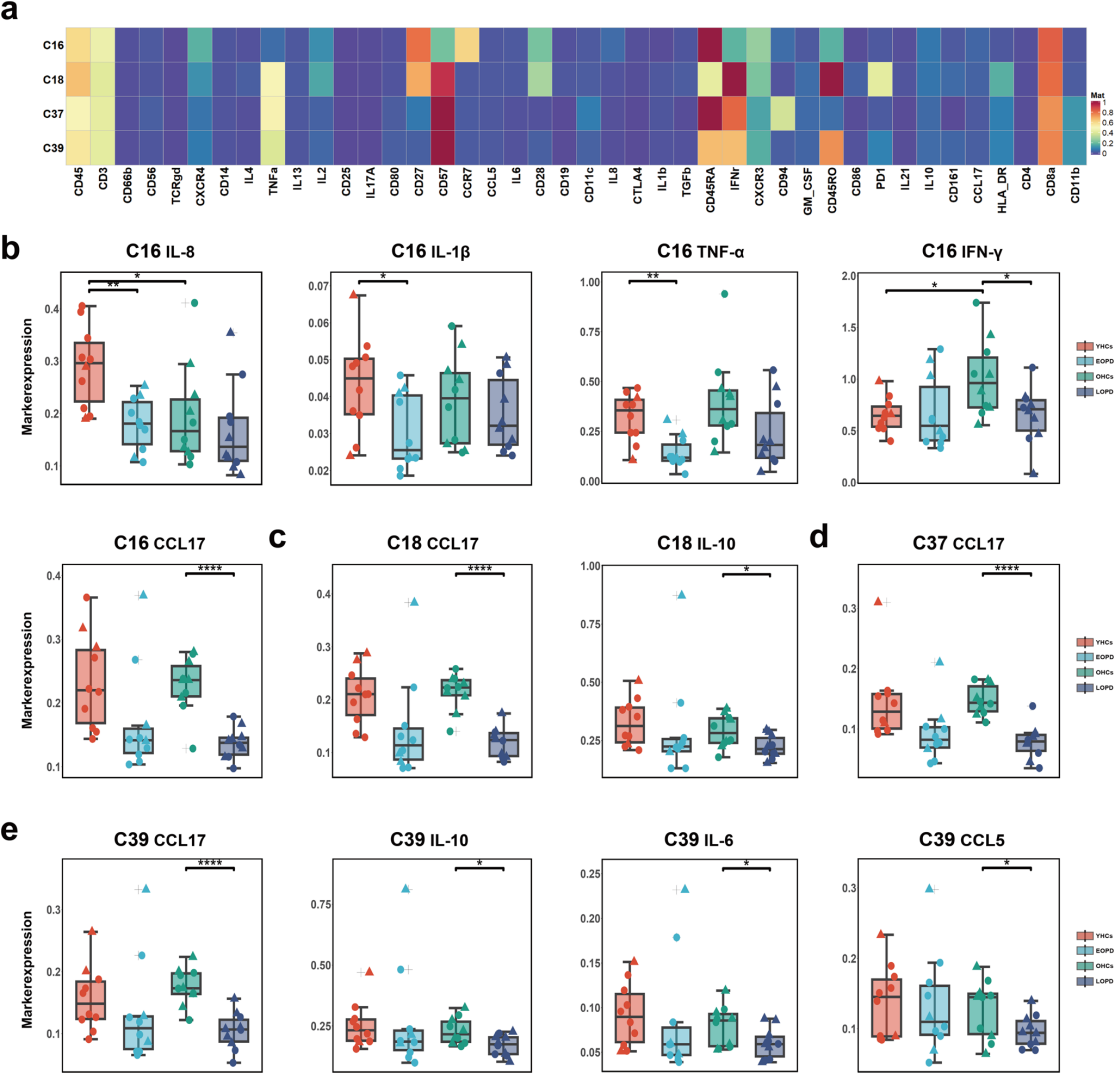
Decreased cytokine and chemokine expression in CD8^+^ T-cell clusters in PD. **a** Heatmap of immune cell marker expression for the four selected CD8^+^ T-cell clusters. **b–e** Comparison of cytokine and chemokine expression in **b** C16, **c** C18, **d** C37, and **e** C39. ANOVA with Bonferroni correction was used to test statistical significance between groups (*p < 0.05, **p < 0.01, ***p < 0.001). Error bars show the mean ± standard error of the mean (SEM). The box-and-whisker plot was presented as median, 1st quartile, and 3rd quartile of the box, the 95th and 5th percentile for the upper and lower whisker, "+" for the outliners. △ = male; ○ = female.

**Fig. 3:**
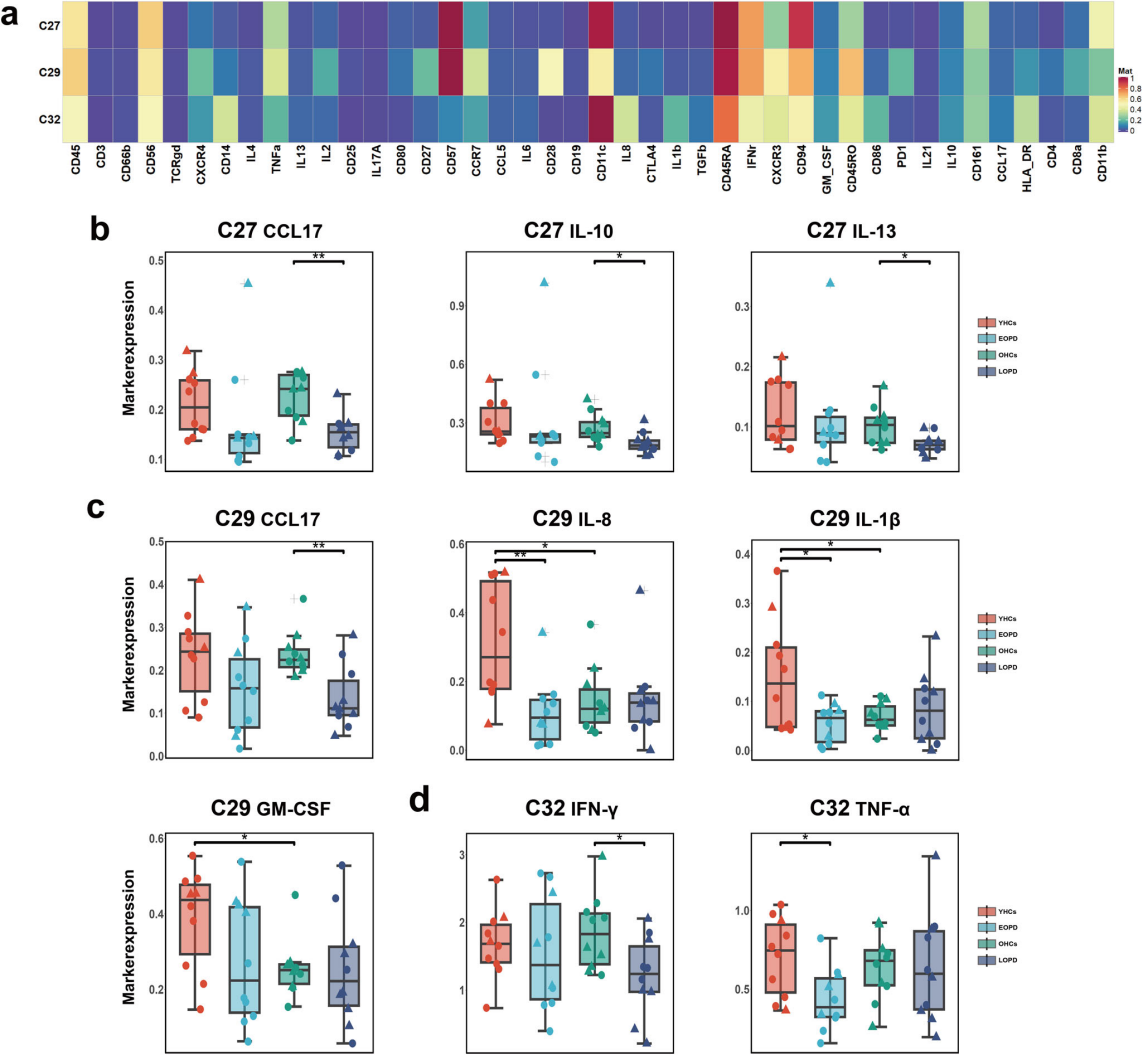
Decreased cytokine and chemokine expression in NK cell clusters in PD. **a** Heatmap of immune cell marker expression for the three selected NK cell clusters. **b–d** Comparison of cytokine and chemokine expression in **b** C27, **c** C29, **d** C32. ANOVA with Bonferroni correction was used to test statistical significance between groups (*p < 0.05, **p < 0.01, ***p < 0.001). Error bars show the mean ± standard error of the mean (SEM). The box-and-whisker plot was presented as median, 1st quartile and 3rd quartile of the box, the 95th and 5th percentile for the upper and lower whisker, "+" for the outliners. △ = male; ○ = female.

Mass cytometry was used to analyze PBMCs of 40 subjects, including 10 EOPD, 10 LOPD, 10 YHCs, and 10 OHCs. We selected the seven representative cell clusters from 60 cell clusters screened using clustering algorithms, t-SNE, and unsupervised clustering. These seven clusters were distinguished based on marker expression patterns. To further characterize previously identified effector CD8^+^ T cells (C16, C18, C37, and C39) and NK cells (C27, C29, and C32)^11^, intracellular cytokine and chemokine expression was evaluated to assess immune function (Fig. 1a). It is worth noting that we chose to detect intracellular cytokines and chemokines after stimulation to efficiently identify the secretory function of single-cell subpopulations. The frequency of immune cells assayed after stimulation was not consistent with our previous study as a large number of cells were lost during stimulation. We, therefore, divided the cytokine and chemokine expression by the cell numbers in this group to exclude interference caused by different cell numbers. The selected clusters belonged to two lineages, including CD8^+^ T cells and NK cells. The four CD8^+^ T-cell clusters included C16 (CD57^−^ naïve CD8^+^ T), C18 (CD8^+^ effector memory T), C37 (CD57^+^ CD8^+^ terminally differentiated effector memory T), and C39 (CD57^+^ CD8^+^ effector memory T). The three NK cell clusters included C27 (CD56^+^ CD16^+^ CD57^+^ CD28^−^), C29 (CD56^+^ CD16^+^ CD57^+^ CD28^+^), and C32 (CD56^+^ CD16^+^ CD57^−^ CD28^−^) (Figs. 2a, 3a, Supplementary Table 1). Normalized abundances in patients with PD and HCs, and the EOPD, LOPD, YHC, and OHC subgroups, were then compared. The results revealed significantly decreased IFN-γ and TNF-α expression in LOPD compared with that in OHCs (Fig. 1b), as well as decreased TNF-α expression in PD compared with that in HCs (Supplementary Fig. 1).

### Decreased cytokine and chemokine expression in CD8^+^ T-cell clusters in PD

Comparison of the subgroups revealed decreased expression of IFN-γ and CC-chemokine ligand (CCL)17 in C16 (Fig. 2b), CCL17 and IL-10 in C18 (Fig. 2c), CCL17 in C37 (Fig. 2d), and CCL17, IL-10, IL-6 and CCL5 in C39 (Fig. 2e) in LOPD compared with those in OHCs. Significantly decreased expression of IL-8, IL-1β, and TNF-α in C16 (Fig. 2b) was seen in EOPD compared with that in YHCs. The HCs also exhibited differences between the two age groups, with the YHCs exhibiting lower IFN-γ expression but higher IL-8 expression in C16 than the OHCs (Fig. 2b). In addition, TNF-α, IL-8, transforming growth factor (TGF)-β, and IL-1β expression were decreased in C16 (Supplementary Fig. 2) compared with those in HCs. CCL17 expression was significantly decreased in C16, C18, C37, and C39 clusters in PD compared with those in HCs (Supplementary Fig. 2).

### Decreased cytokine and chemokine expression in NK cell clusters in PD

The results revealed significantly decreased expression of CCL17, IL-10, and IL-13 in C27 (Fig. 3b); CCL17 in C29 (Fig. 3c); and IFN-γ in C32 in LOPD (Fig. 3d) compared with those in OHCs, as well as significantly decreased expression of IL-8 and IL-1β in C29 (Fig. 3c) and TNF-α in C32 in EOPD (Fig. 3d) compared with those in YHCs. The HCs also exhibited differences between the two age groups, with the YHCs exhibiting significantly higher expression of IL-8, IL-1β, and granulocyte-macrophage colony-stimulating factor (GM-CSF) in C29 (Fig. 3c) than the OHCs. The three NK cell clusters also exhibited decreased cytokine and CCL17 expression in patients with PD (Supplementary Fig. 3). In addition, expression of IFN-γ, TNF-α, and IL-8 in C29 and GM-CSF in C32 was significantly decreased in PD (Supplementary Fig. 3) compared with that in HCs.

### Specific cytokines and chemokines associated with clinical features

**Fig. 4:**
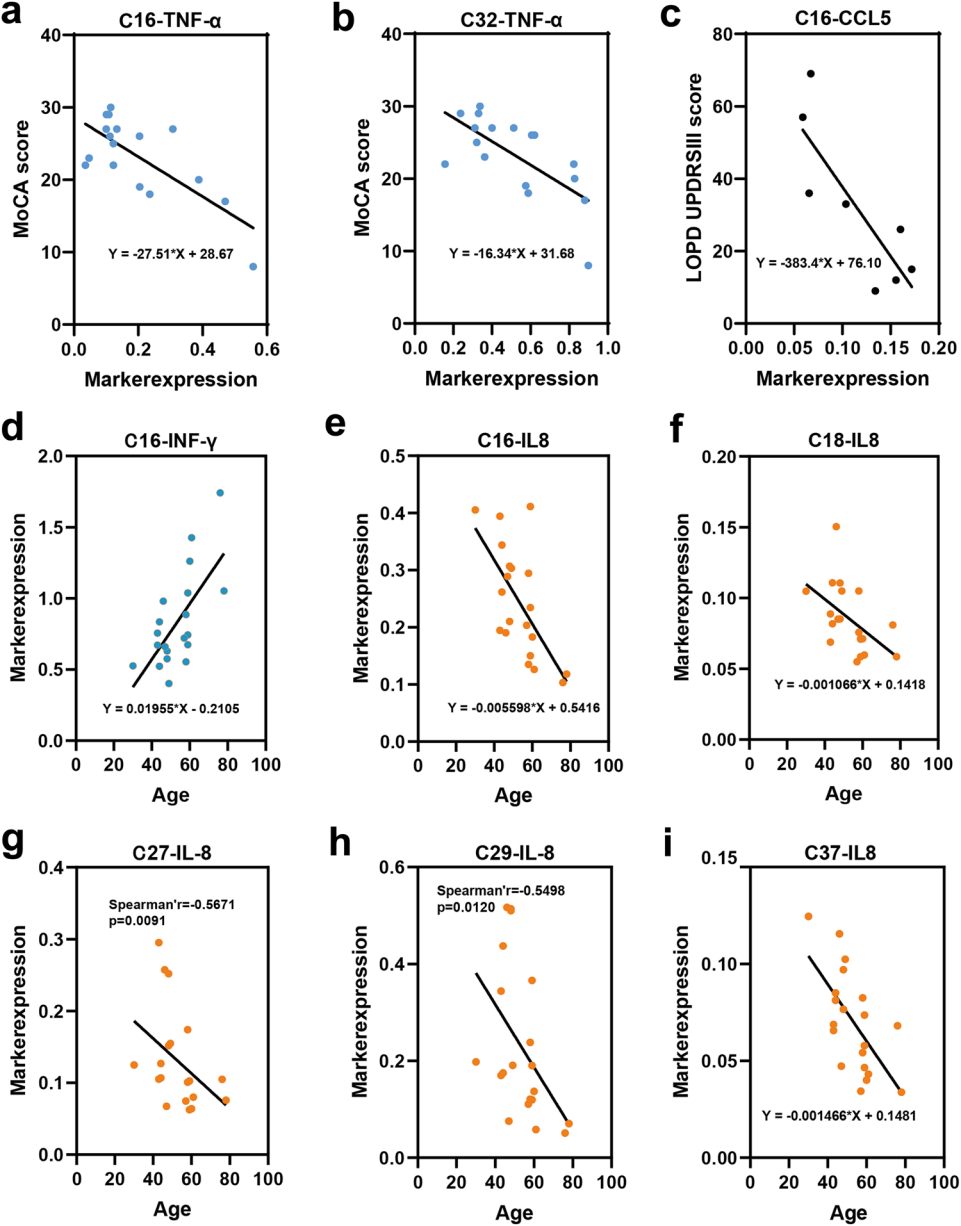
Correlations between cytokine and chemokine expression and clinical characteristics, including age, MoCA score, and UPDRS III score. **a** and **b** MoCA scores decrease with increased TNF-α expression in **a** C16 (*p* = 0.001, *R*^2^ = 0.540), and **b** C32 (*p* = 0.002, *R*^2^ = 0.473). **c** UPDRS III scores decrease in LOPD with increased CCL5 expression in C16 (*p* = 0.011, *R*^2^ = 0.689). **d** IFN-γ expression in C16 increases with age (*p* = 0.001, *R*^2^ = 0.442). IL-8 expression decreases with age in **e** C16 (*p* = 0.002, *R*^2^ = 0.429), **f** C18 (*p* = 0.02, *R*^2^ = 0.264), **g** C27 (*p* = 0.009, rho = 0.567), **h** C29 (*p* = 0.012, rho = 0.550), and **i** C37 (*p* = 0.002, *R*^2^ = 0.408). Bivariate correlation and linear regression were used to access associations between variables and demographic features, adjusted for relevant confounders, including age, duration, and sex. MoCA Montreal cognitive assessment, UPDRS unified Parkinson’s disease rating scale.

Motor and cognitive function in patients with PD was assessed using UPDRS, MMSE, and MoCA scores, and correlated with cytokine and chemokine expression. The results indicated that expression of TNF-α, an important pro-inflammatory cytokine^12^, in C16 and C32 is negatively correlated with MoCA scores (Fig. 4b), indicating that elevated TNF-α in C16 and C32 clusters might be associated with cognitive function impairment. Multivariate stepwise linear regression identified significant correlations between UPDRS III scores in patients with PD and two independent variables, TNF-α and IFN-γ, in C16. TNF-α expression was positively correlated with UPDRS III scores, whereas IFN-γ expression was negatively correlated with UPDRS III scores (Table 1). Additionally, for the LOPD group, a negative linear correlation was observed between CCL5 expression in C16 and UPDRS III scores (Fig. 4c). Although CCL17 expression was significantly decreased in all seven clusters in patients with PD (Supplementary Figs. 2 and 3), no relationship was observed between CCL17 expression and clinical characteristics.

**Table 1:** Baseline demographic and clinical characteristics of the four groups.

	YHCs (*n* = 10)	OHCs (*n* = 10)	EOPD (*n* = 10)	LOPD (*n* = 10)	Range at BL	*P* value
Age (yr)	44.20 ± 5.45	62.50 ± 7.74	41.34 ± 6.20	65.04 ± 7.25	–	<0.001^a^
Sex (F/M)	8/2	5/5	7/3	4/6	–	0.31^b^
Age of onset (yr)	–	–	39.23 ± 6.65	63.13 ± 7.71	–	<0.001^c^
Disease duration (yr)	–	–	2.11 ± 1.70	1.91 ± 1.39	–	0.78^d^
H–Y stage	–	–	1.83 ± 0.50	2.31 ± 1.00	–	0.34^c^
UPDRS-Total	–	–	28.67 ± 12.60	49.00 ± 34.92	15–114	0.16^d^
UPDRS-III	–	–	18.56 ± 9.33	32.13 ± 21.60	8–69	0.11^d^
MMSE	–	–	28.11 ± 2.42	24.00 ± 5.78	12–30	0.05^c^
MoCA	–	–	25.33 ± 4.00	20.88 ± 6.45	8–30	0.10^d^

All values are expressed as mean ± SD. Error bars show the mean ± standard error of the mean (SEM).

*OHCs* older adult healthy controls, *EOPD* early onset-PD, *LOPD* late onset-PD, *YHCs* young healthy controls, *UPDRS* unified Parkinson’s disease rating scale, *H–Y* Hoehn–Yahr, *MoCA* Montreal cognitive assessment.

^a^Kruskal–Wallis test.

^b^Fisher’s precision probability test.

^c^Wilcoxon Mann–Whitney test.

^d^Two-sided *t*-test.

Many cytokines exhibited significantly higher expression in clusters of YHCs than in clusters of OHCs, which suggests a trend of decreasing cytokine expression with age in healthy populations. Bivariate correlation analysis revealed a significant negative relationship between IL-8 expression in C16, C18, C27, C29, and C37 and age in HCs (Fig. 4e–i). In contrast, IFN-γ expression in C16 showed a significant positive correlation with age in HCs (Fig. 4a). These correlations were not seen for patients with PD.

## Discussion

In this study, we found impaired cytokine secretion by CD8^+^ T and NK cells in patients with PD compared with that in HCs, especially in patients with LOPD. These cell subpopulations are, therefore, not only impaired in number, as previously reported^11^, but also in their cytokine and chemokine secretion ability, indicating consistent immune impairment in patients with PD. These results seem to contradict the prevailing view that PD is an immune-activating disease^3^, with studies reporting increased peripheral cytokine or chemokine levels in patients with PD^13–16^. This may be explained by the detection of intracellular cytokine and chemokine expression after stimulation, which allowed evaluation of the secretory function of single-cell subpopulations rather than overall secretion levels in serum. Furthermore, this study selected drug-naïve patients, most of whom were in the early stages of PD, which may be indicative of an immunosuppressive state specific to the onset of PD.

TNF-α and IFN-γ expression in naïve CD8^+^ T-cell subsets (C16) correlated with UPDRS III scores. Recent studies have revealed reduced naïve CD8^+^ T cells (CD3^+^ CD8^+^ CD45RA^+^ CD45RO^−^) in patients with PD, and a positive correlation between the proportion of naïve CD8^+^ T cells and the severity of autonomic dysfunction and psychiatric comorbidity, but a negative correlation between the severity of sleep behavior disorders and rapid eye movement^17^. These results suggest that the role of CD8^+^ T cells in PD requires further investigation, and especially naïve CD8^+^ T cells may be candidates for clinical and pathogenetic studies in PD.

The role of NK cells in PD has received increasing attention. Recently, studies found that NK cell depletion results in increased phosphorylated α-synuclein deposits in a preclinical mouse model of PD, suggesting a potential protective role for NK cells^18^. Moreover, α-synuclein aggregates attenuated NK cell cytotoxicity and decreased IFN-γ release in vitro^18^. NK cells can secrete IFN-γ or TNF-α to kill target cells. Interestingly, synovial NK cells produce GM-CSF and promote neutrophil infiltration in persistent inflammatory arthritis^19^. Similarly, we found that NK cells from patients with PD were able to secrete GM-CSF, but the expression of GM-CSF in C32 was reduced in PD. We, therefore, hypothesize that GM-CSF in NK cells plays a vital role in PD.

Our investigation of chemokines revealed a prominent role for CCL17 in PD. As a chemoattractant, CCL17 is mainly involved in the recruitment of CD4^+^ Tregs and Th17 cells, while also inhibiting Treg cell expansion and promoting differentiation of Th1 and Th17 cells through the secretion of IL-12 and IL-23 by dendritic cells^20^. Increased CCL17 expression has been observed in the frontal cortex of patients with Alzheimer’s disease^21^ and in the CSF of patients with multiple sclerosis^22^. Furthermore, in a mouse model of subarachnoid hemorrhage, increased CCL17 expression was observed in neurons after oxyhemoglobin stimulation, which can improve neurological functions by promoting M2-like polarization of microglia^23^. Consistent with these reports, our results revealed reduced CCL17 expression in the peripheral blood in all seven tested clusters. However, the effects of CCL17 on T cells and NK cells remain unknown, necessitating further studies to verify CCL17 expression in peripheral blood, CSF, and central nervous system tissue samples in PD and to clarify the role and mechanism of action of CCL17.

The present study had several limitations. First, although we made every effort to exclude interference from drugs^24^, disease states, and infections, the immune status across different populations remains heterogeneous, which may impact results, especially with our small sample size and sex imbalance in EOPD and YHCs. Second, some of the LOPD patients had low scores on MMSE or MoCA because of low education levels and cultural differences, which may influence the judgement of cognitive level in this population. Furthermore, PD patients were not tested for mutations, especially some genes that are really relevant with inflammation. Third, although CyTOF data can be highly sensitive, more data are required to limit the effect of false positives^25^. Another limitation is that the cross-sectional nature of our study is suggestive rather than confirmatory. Future studies should aim to isolate immune cells of interest within each subpopulation to verify their function and potential mechanism with a larger sample size and longitudinal follow-up in PD.

It is worth noting that sex has an impact on the immunological response. Healthy males were found to have higher CD8^+^ T cell and NK cell frequencies than females, while women had higher numbers of activated CD8^+^ T cells in peripheral blood following in vitro stimulation of PBMCs^26^. In PD patients, studies have found CD14^+^ HLA-DR^+^ monocytes were higher in male than in female patients^27^. Another study showed that the percentage of CD4^+^ T cells in women was inversely correlated with the H&Y stage, and IgG levels were positively correlated with disease duration and UPDRS III scores^28^. However, these correlations did not exist in male patients. Overall, peripheral immunization studies should include large and sex-balanced samples, and pay attention to differences between the sexes.

Some genes also influence the peripheral immune status in PD. A small percentage of sporadic PD, particularly EOPD, also contains genetic mutations. Recently, a large cohort study of a sporadic EOPD population in the Chinese mainland identified variants of PD-associated genes in 57 of 1242 (4.59%), primarily *PRKN* (2.65%) and leucine-rich repeat kinase 2 (*LRRK2*, 0.4%)^29^. *LRRK2*, the second most common mutation in sporadic EOPD, has been found to be associated with peripheral immune response. It has been discovered that *LRRK2*-associated familial PD patients have higher levels of pro-inflammatory cytokines, including IL-1β, TNF-a, IL-12, and IL-6^30^. In addition, the expression of *LRRK2* was found to be increased in T cells, and there was a positive correlation between *LRRK2* expression and IFN-γ, TNF, and IL-2 expression in T cells^31^. However, a recent study found that familial PD patients with *LRRK2* mutations did not show significant differences in the lymphocyte or the neutrophil count compared to HCs^32^. Conversely, glucocerebrosidase 1 (*GBA*)-associated familial PD, as well as sporadic PD, showed a significantly lower lymphocyte count than HCs^32^. The effect of *PRKN* on the peripheral immune remains unclear, which appears to be mediated through mitochondrial antigens^3^.

In conclusion, we found reduced cytokine and chemokine expression in seven subpopulations of CD8^+^ T cells and NK cells in PD compared with HCs. When considered together with our previous report of decreased effector CD8^+^ T cells and lower cytotoxicity in NK cells, we hypothesize that peripheral immunity is at least partially suppressed in drug-naïve patients with PD. In addition, our results highlight that CCL17 expression is decreased in all seven tested clusters, suggesting that CCL17 has an important role in PD. Our results revealed a more detailed cytokine and chemokine map of peripheral specific CD8^+^ T and NK cell subsets, as well as disrupted secretory function in patients with PD. These results further our understanding of the peripheral immune response in PD and provide valuable insight for further mechanistic investigation.

## Methods

### Subjects

Ten patients with early-onset PD (EOPD; ≤50 years) and 10 patients with late-onset PD (LOPD; >50 years of age) participated in this study. All patients were recruited from the neurology department of the Second Affiliated Hospital of Zhejiang University and diagnosed by senior movement disorder specialists based on current diagnostic criteria^33^. All patients’ family histories of PD were excluded, and dementia was ruled out by asking about the patient’s medical history, such as their ability to take care of themselves. To confirm the diagnosis of idiopathic PD, all patients underwent clinical follow-up after one year. Likewise, 10 young healthy controls (YHCs) and 10 older adult healthy controls (OHCs) were recruited based on age and sex (Table 2). Patients with other neurodegenerative diseases, tumors, infections, immune system diseases, antibiotic or immunosuppressive treatment within 1 month, and vaccination within 3 months were excluded. Recruited patients were all drug naïve. Data concerning clinical characteristics, including the age of onset, disease duration, Hoehn and Yahr (H&Y) stage, UPDRS III, Montreal cognitive assessment (MoCA), and MMSE scores (Table 1 and Supplementary Table 4), and peripheral blood samples were collected.

**Table 2:** Multivariate stepwise linear regression analysis, with UPDRS III score as the dependent variable.

Variable	*β* coefficient	*t*	*P*-value	*F*	Adjusted *R*^2^
C16 TNF-α	−0.507	−2.170	0.048	6.499^**^	0.407
C16 IFN-γ	0.841	3.602	0.003		

Multivariate stepwise linear regression analysis was used to explore relationships between cytokines and UPDRS III score.

*TNF* tumor necrosis factor; *IFN* interferon.

**p < 0.01

### Standard protocol approvals, registrations, and patient consent

Ethical approval was obtained through the Medical Ethics Committee of the Second Affiliated Hospital of Zhejiang University School of Medicine (2020-596). All patients and HCs provided written informed consent.

### Sample processing for mass cytometry

Using a 10 mL pipette, 10 mL blood samples were transferred to 50 mL centrifuge tubes and diluted to 20 mL with phosphate-buffered saline (PBS; GENOM). The diluted sample was slowly added to the top layer of 10 mL Ficoll separation solution (GE Healthcare) in a 50 mL centrifuge tube without disrupting the upper liquid level and subsequently centrifuged at 400 × *g* for 15 min in a centrifuge with a plate rotor (Avanti J-15R, Beckman). To prevent peripheral blood mononuclear cell (PBMC) loss, 0.5 mL of the waste liquid above the separated white film layer was reserved. The Ficoll layer was repeatedly aspirated with a 1 mL manual pipette until no visible cells were detected in the white layer. FACS buffer was added to the initial PBMC extract to a final volume of 30 mL, centrifuged for 10 min at 400 × *g*, and the supernatant discarded. After adding 1 mL ammonium-chloride–potassium (ACK) lysing buffer to the cells, the mixture was blown, mixed, and allowed to stand for 2 min. FACS buffer was then added to the PBMC suspension to a final volume of 10 mL, centrifuged at 400 × *g* for 5 min, and the supernatant discarded. The precipitate was resuspended in 4 mL FACS buffer. Trypan blue (Solarbio) was added to10 µL cell suspension for cell counting. The mixture was centrifuged at 400 × *g* for 5 min, the supernatant was discarded, and the cell precipitate was collected.

### Stimulation of blood cell populations for cytokine and chemokine responses

As <1% of non-stimulated cells produce cytokines and chemokines, ex vivo cell activation was required to induce cytokine and chemokine production. Cells were resuspended to 5 × 10^6^ cells/mL in RPMI1640 medium supplemented with 10% fetal bovine serum (FBS) and 1% penicillin/streptomycin (P/S). eBioscience™ cell stimulation cocktail (500X) was added into the culture medium at a ratio of 2 µL–1 mL cell suspension, lightly blown and mixed, and incubated at 37 °C, 5% CO_2_ for 4 h. The stimulation cocktail contained a mixture of phobolol ester 12-tetradecanoate 13-acetate (PMA), ionomycin, Brefeldin A, and monensin. PMA and ionomycin induce cytokine and chemokine production in most cell types, whereas Brefeldin A and monensin induce the accumulation of secretory proteins in the endoplasmic reticulum and Golgi apparatus. Cell suspensions were collected into centrifuge tubes, and culture vessels were washed with pre-warmed (37 °C) RPMI 1640 + 10% FBS + 1% P/S medium and collected into centrifuge tubes to ensure recovery of all cells. Finally, cell suspensions were centrifuged at 37 °C at 400 × *g* for 5 min to obtain cell precipitates for counting. Cell recovery was typically between 35% and 50%.

### Mass cytometry staining and data acquisition

Cells were washed once with PBS to remove dead cells and stained with 100 μL of 250 nM cisplatin (Fluidigm) on ice for 5 min. After incubating in an Fc-receptor-blocking solution, cells were stained with a surface-antibody cocktail (Supplementary Tables 2 and 3) on ice for 30 min. After two washes with FACS buffer (PBS + 0.5% BSA), the cells were fixed with 200 μL intercalation solution (Maxpar Fix and Perm Buffer containing 250 nM 191/193 Ir; Fluidigm) overnight. After washing once with FACS buffer and once with permeabilization buffer (eBioscience™), cells were stained with intracellular antibodies (Supplementary Tables 2 and 3) on ice for 30 minutes. The cells were then washed and resuspended in deionized water, added to 20% EQ beads (Fluidigm), and analyzed on a mass cytometer (Helios, Fluidigm).

### Cytometry by time of flight (CyTOF) data analysis

A doublet-filtering scheme was used to decode the data from raw data tagged with unique barcodes^34^. The beads normalization method was used to normalize FCS files generated by different batches^35^. Manual gating using FlowJo software allowed the retention of only live, single immune cells, excluding debris, dead cells, and doublets. Cells were partitioned based on marker expression level using the X-shift clustering algorithm^36^. The cell types of each cluster were annotated according to the marker expression pattern on a heatmap of clusters versus markers. T-distributed stochastic neighbor embedding (t-SNE), a dimensionality reduction algorithm, was used to visualize high-dimensional data in two dimensions and to show the distribution of each cluster and differences between groups^37^.

### Statistical analyses

Data analyses were performed using SPSS version 25.0 (Armonk, NY, IBM Corp) and GraphPad Prism 9 (San Diego, CA). Comparisons of intracellular cytokines and chemokines among all four subgroups (YHCs, EHCs, EOPD, and LOPD) were performed by one-way ANOVA analysis, and Bonferroni correction was used as a multiple comparison adjustment. Additionally, a two-sided *t*-test statistical analysis was used to test the difference between the two groups. Multivariate stepwise regression was used to identify factors associated with clinical characteristics, including UPDRS, UPDRS III, MoCA, and MMSE scores of patients with PD. Bivariate correlation and linear regression were used to access associations between variables and demographic features, adjusted for relevant confounders, including age, duration, and sex. Statistical significance was considered as *p* < 0.05.

### Reporting summary

Further information on research design is available in the Nature Research Reporting Summary linked to this article.

## Supplementary information

Supplementary Files

Reporting Summary

## Data Availability

Anonymized data not published within this article will be made available upon request.
