# Supplementary material for: Cytokine and chemokine map of peripheral specific immune cell subsets in Parkinson’s disease
Source: NPJ Parkinsons Dis. 2023 Jul 25;9:117. doi: 10.1038/s41531-023-00559-0 (PMC10368737; doi:10.1038/s41531-023-00559-0)
Supplement: Supplementary file 1 — Supplementary Files [file 41531_2023_559_MOESM1_ESM.pdf]

Supplementary Figures with legends beneath each one

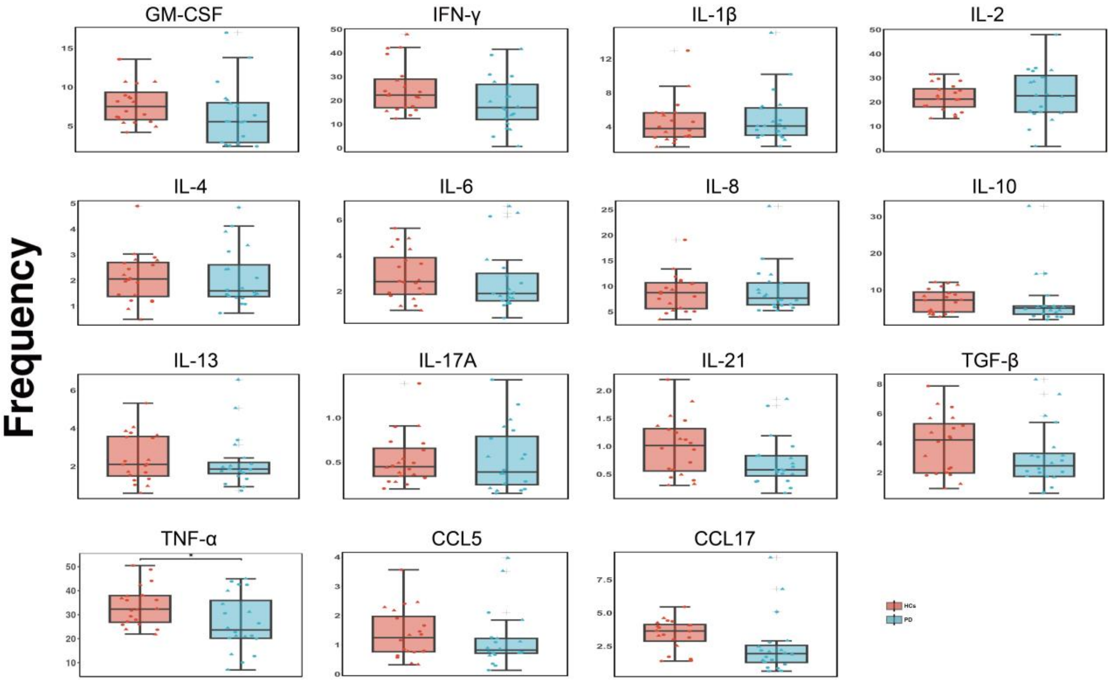

Supplementary Fig. 1 Differences in cytokine and chemokine expression in specific immune cell subsets between PD and HCs. Two-sided *t* tests were used to test statistical significance between groups (**p*<0.05, ***p*<0.01, ****p*<0.001). Error bars show the mean ± SEM. △=male； ○=female. The box-and-whisker plot was presented as median, 1st quartile and 3rd quartile of the box, the 95th and 5th percentile for the upper and lower whisker, "+" for the outliners.

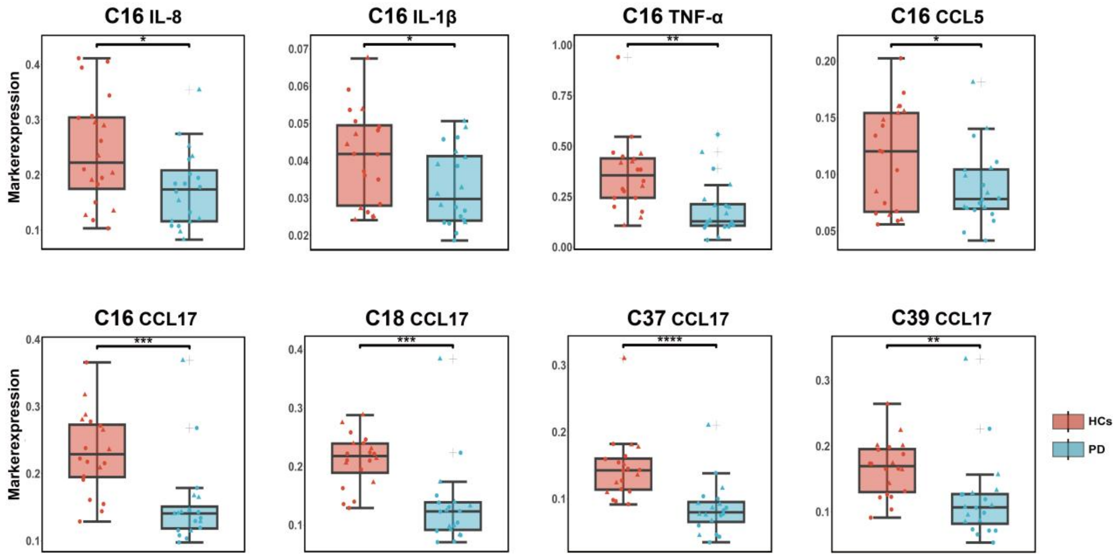

Supplementary Fig. 2 Decreased cytokine and chemokine expression in CD8^{+} T-cell clusters in PD. Two-sided *t* tests were used to test statistical significance between groups (**p*<0.05, ***p*<0.01, ****p*<0.001). Error bars show the mean ± SEM. △=male；○=female. The box-and-whisker plot was presented as median, 1st quartile and 3rd quartile of the box, the 95th and 5th percentile for the upper and lower whisker, "+" for the outliners.

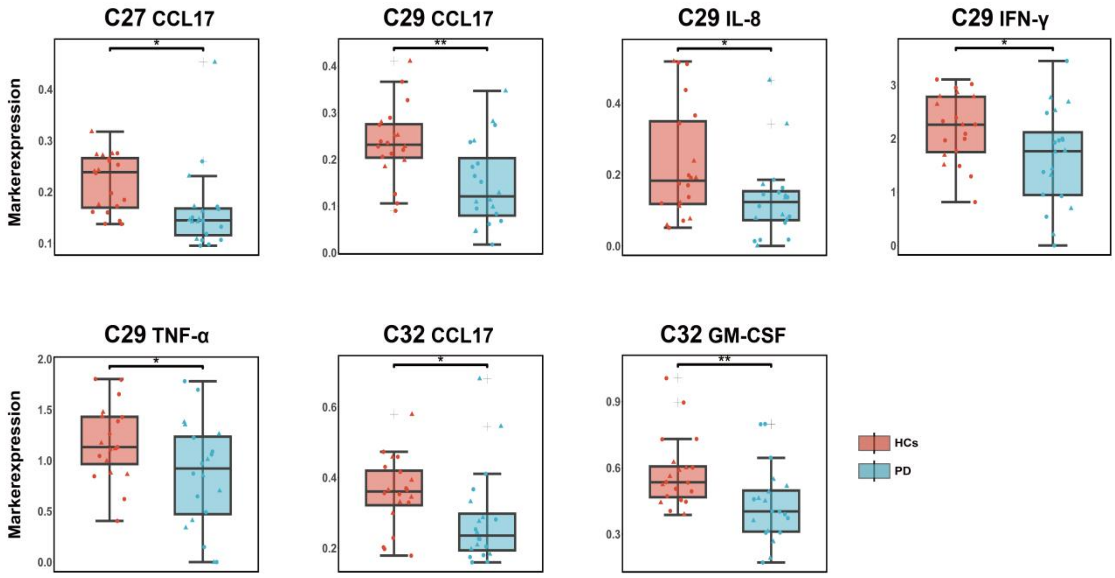

Supplementary Fig. 3 Decreased cytokine and chemokine expression in NK cell clusters in PD. Two-sided *t* tests were used to test statistical significance between groups (**p*<0.05, ***p*<0.01, ****p*<0.001). Error bars show the mean ± SEM. △=male； ○=female. The box-and-whisker plot was presented as median, 1st quartile and 3rd quartile of the box, the 95th and 5th percentile for the upper and lower whisker, "+" for the outliners.

Supplementary Tables

eTable 1: List of lineages and subpopulations derived from PBMCs

Lineage	Cluster	Subpopulation	Markers
$CD8^+$ T cells	C16	Naïve $CD8^+$ T cells	CD3+ CD56- TCRgd- CD8+ CD45RO- CD197+
	C18	$CD8^+$ T_{EM} cells	CD3+ CD56- TCRgd- CD8+ CD45RO+ CD197- CD57+ CD27+ CD94-
	C37	$CD8^+$ T_{EMRA} cells	CD3+ CD56- TCRgd- CD8+ CD45RO- CD197- CD57+ CD161-
	C39	$CD8^+$ T_{EM} cells	CD3+ CD56- TCRgd- CD8+ CD45RO+ CD197- CD57+ CD27- CD94-
NK cells	C27	CD57+ CD28- NK cells	CD3- CD19- CD56+ CD57+ CD28- CD11b+ CD11c+
	C29	CD57+ CD28+ NK cells	CD3- CD19- CD56+ CD57+ CD28+
	C32	CD57- CD28- NK cells	CD3- CD19- CD56+ CD57- CD28- CD11c+

Abbreviations: T_{CM} = central memory T cells; T_{EM} = effector memory T cells; T_{EMRA} = terminally differentiated effector memory re-expressing CD45RA T cells; NK = natural killer.

eTable 2: List of surface markers and intracellular cytokines/chemokines markers for a quick search

Surface Markers	
CD45	CXCR4
CD3	CXCR3
CD4	CD57
CD8a	CD19
TCRγδ	CD56
CD45RO	CD80
CD45RA	CD86
CCR7	CD11b
CD25	CD66b
CD152	CD14
CD27	CD11c
CD28	HLA-DR
PD-1	CD94
CD161	

Cytokines/Chemokines
IL-1β
IL-2
IL-4
IL-6
IL-8
IL-10
IL-13
IL-17A
IL-21
IFN-γ
GM-CSF
TNF-α
TGF-β
CCL5
CCL17

eTable 3: Antibodies used for mass cytometry and the surface marker being detected

List	Label	Markers	Clone	Company
1	89Y	CD45	HI30	Biolegend
2	115ln	CD3	UCHT1	Bio x cell
3	139La	CD66b	G10F5	Biolegend
4	141Pr	CD56	NCAM16.2	BD biosciences
5	142Nd	TCR γ/δ	5A6.E9	Thermofisher
6	143Nd	CD184(CXCR4)	12G5	Biolegend
7	144Nd	CD14	M5E2	Biolegend
8	145Nd	IL-4	MP4-25D2	Biolegend
9	146Nd	TNF-α	MAb11	Biolegend
10	147Sm	IL-13	JES10-5A2	Biolegend
11	148Nd	IL-2	MQ1-17H12	Biolegend
12	149Sm	CD25(IL-2Rα)	24212	R&D
13	150Nd	IL-17A	BL168	Biolegend
14	151Eu	CD80(B7-1)	2D10.4	eBioscience
15	152Sm	CD27	O323	Biolegend
16	153Eu	CD57	HNK-1	Biolegend
17	154Sm	CD197(CCR7)	G043H7	Biolegend
18	155Gd	CCL5(RANTES)	21445	R&D
19	156Gd	IL-6	MQ2-13A5	Biolegend
20	157Gd	CD28	CD28.2	Biolegend
21	158Gd	CD19	HIB19	Biolegend
22	159Tb	CD11c	Bu15	Biolegend
23	160Gd	IL-8	E8N1	Biolegend
24	161Dy	CD152 (CTLA-4)	BN13	Bio x cell
25	162Dy	IL-1 β(IL-1F2)	8516	R&D
26	163Dy	LAP(TGF-β1)	TW7-28G11	Biolegend
27	164Dy	CD45RA	HI100	Biolegend
28	165Ho	IFN-γ	B27	Biolegend
29	166Er	CD183(CXCR3)	G025H7	Biolegend
30	167Er	CD94	HP-3D9	BD biosciences
31	168Er	GM-CSF	BVD2-21C11	Biolegend
32	169Tm	CD45RO	UCHL1	Biolegend
33	170Er	CD86	FUN-1	BD biosciences
34	171Yb	CD279(PD-1)	EH12.2H7	Biolegend
35	172Yb	IL-21	3A3-N2	Biolegend
36	173Yb	IL-10	JES3-9D7	Biolegend
37	174Yb	CD161	HP-3G10	Biolegend
38	175Lu	CCL17(TARC)	54015	R&D
39	176Yb	HLA-DR	L243	BioLegend

40	197Au	CD4	RPA-T4	BioLegend
41	198Pt	CD8a	RPA-T8	BioLegend
42	209Bi	CD11b	M1/70	BioLegend

eTable 4: Clinical information about the EOPD and LOPD patients

Subject	Onset	Gender	Age	drinking or not	smoking or not	family history	medications	Onset with tremor or not	Onset of disease
1	Early	M	43.05	drinking for 30 years	Smoking for 6 years, 10 sticks/day, but has stopped for 20 years	N	N	Y	Left upper limb
2	Early	F	35.49	N	N	N	N	N	Right upper limb
3	Early	M	37.73	N	Smoking for 25 years, 10 sticks/day	N	N	N	Left upper limb
4	Early	F	30.00	N	N	N	N	N	Unclear
5	Early	F	40.81	N	N	N	N	Y	Left upper limb
6	Early	F	41.05	N	N	N	N	N	Left upper limb
7	Early	F	39.47	N	N	father has the history of PD	N	Y	Left upper limb
8	Early	M	46.70	drinking for 10 years	Smoking for 25 years, 10 sticks/day	N	N	N	Left limb

9	Early	F	49.62	N	N	N	N	Y	left limb
10	Early	F	49.49	N	N	N	N	Y	Right upper limb
11	Late	F	68.79	N	N	N	N	Y	Right limb
12	Late	F	57.12	N	N	N	N	Y	Right upper limb
13	Late	M	58.83	N	N		N	Y	Unclear
14	Late	M	59.80	N	Smoking for 30 years, 40 sticks/day, but has stopped for half a year	N	N	Y	Left upper limb
15	Late	M	70.00	N	N	N	N	N	Unclear
16	Late	F	58.07	N	N	N	N	Y	Left upper limb
17	Late	M	71.00	N	N	N	N	N	Unclear
18	Late	M	59.12	drinking for 30 years	Smoking for 40 years, 10 sticks/day	his father has the history of tremor, but not making a definite diagnosis	N	Y	Right upper limb
19	Late	F	77.03	N	N	N	N	Y	Right upper limb

20	Late	M	71.10	drinking for 60 years	Smoking for 10 years, 20 sticks/day, but has stopped for 40 years	N	N	Y	Right upper limb

Abbreviations: EOPD = early-onset PD; LOPD = late-onset PD; M = male; F = female; N = No; Y = Yes.
